# Progranulin-associated primary progressive aphasia: A distinct phenotype?

**DOI:** 10.1016/j.neuropsychologia.2009.09.017

**Published:** 2010-01

**Authors:** Jonathan D. Rohrer, Sebastian J. Crutch, Elizabeth K. Warrington, Jason D. Warren

**Affiliations:** Dementia Research Centre, Department of Neurodegenerative Disease, UCL Institute of Neurology, University College London, UK

**Keywords:** Primary progressive aphasia, Dementia, Progranulin, Aphasia, Language

## Abstract

The neuropsychological features of the primary progressive aphasia (PPA) syndromes continue to be defined. Here we describe a detailed neuropsychological case study of a patient with a mutation in the progranulin (*GRN*) gene who presented with progressive word-finding difficulty. Key neuropsychological features in this case included gravely impoverished propositional speech with anomia and prolonged word-finding pauses, impaired speech repetition most marked for sentences, and severely impaired verbal (with preserved spatial) short-term memory. There was a dissociated profile of performance on semantic processing tasks: visual semantic processing was intact, while within the verbal domain, verb comprehension was impaired and processing of nouns was intact on tasks requiring direct semantic processing but impaired on tasks requiring associative or inferential processing. Brain MRI showed asymmetric left cerebral atrophy particularly affecting the temporo-parietal junction, supero-lateral temporal and inferior frontal lobes. This case most closely resembles the PPA syndrome known as the logopenic/phonological aphasia variant (LPA) however there were also deficits of grammar and speech repetition suggesting an overlap with the progressive non-fluent aphasia (agrammatic) variant (PNFA). Certain prominent features of this case (in particular, the profile of semantic impairment) have not been emphasised in previous descriptions of LPA or PNFA, suggesting that *GRN* may cause an overlapping PPA syndrome but with a distinctive cognitive profile. This neuropsychological evidence suggests that *GRN*-PPA may result from damage involving the temporo-parietal junction and its functional connections in both the dorsal and ventral language networks, with implications for our understanding of language network pathophysiology.

## 1 Introduction

Progressive language impairment as a primary feature of neurodegenerative disease was initially described by Pick (1892) in the late 19th century and such cases continued to be described intermittently in the early 20th century. However, recent decades have seen a resurgence of research in this field. In a series of studies, Mesulam (1982, 2001, 2003) described a group of patients with “primary progressive aphasia” (PPA) who had a variety of different impairments of language. Independently, in the mid 1970s Warrington (1975) described patients with progressive impairment of semantic memory, which was later to be called semantic dementia (SD) (Hodges, Patterson, Oxbury, & Funnell, 1992; Snowden, Goulding, & Neary, 1989). Although language impairment dominated the presentation in these groups it was observed that many of these patients developed behavioural features similar to frontotemporal dementia and hence in the “Neary criteria” of 1998 (Neary et al., 1998) the term ‘frontotemporal lobar degeneration’ (FTLD) was introduced to cover three disorders—the behavioural syndrome of frontotemporal dementia (FTD or behavioural variant FTD, bvFTD) and two syndromes presenting with language impairment: progressive non-fluent aphasia (PNFA), a disorder of speech production with agrammatism, and SD, a disorder of semantic knowledge which commonly presents with fluent aphasia and loss of vocabulary. However, it has long been recognised that a number of patients exhibit language syndromes that do not fit clearly into either the PNFA or SD category as originally defined, particularly in the non-fluent aphasia category which is more heterogeneous. More recently, Gorno-Tempini et al. (2004, 2008) have described a third syndrome designated the “logopenic/phonological variant” of PPA (LPA) and characterized by slow speech rate with long word-finding pauses and impaired verbal short-term memory. These authors proposed modified criteria for “PNFA” emphasizing the motor speech impairment (apraxia of speech) and agrammatism. This tripartite division of the PPA spectrum underlines the inadequacy of the fluent/non-fluent dichotomy as a descriptor of progressive aphasias. However, definition of the LPA syndrome remains challenging, and ‘logopenia’ is itself a clinical descriptor which requires further neuropsychological analysis.

Until recently, descriptions of the PPA syndromes had been purely clinical but recent genetic and pathological studies have shed light on the molecular basis of PPA. In the majority of studies, SD is chiefly associated with TDP-43 pathology (Davies et al., 2005; Snowden, Neary, & Mann, 2007a). PNFA is more frequently associated with tau-positive pathology at post-mortem (Josephs et al., 2006; Knibb, Xuereb, Patterson, & Hodges, 2006) however non-tau pathologies are well documented (e.g., Josephs, Stroh, Dugger, & Dickson, 2009; Shi et al., 2005): it has been proposed that patients with motor speech impairment are more likely to have tau pathology while those without motor speech impairment may be more likely to have TDP-43 pathology (Josephs, 2008). There are currently few studies of LPA with histopathological correlation, however early work has emphasised an association with Alzheimer's disease (AD) pathology (Mesulam et al., 2008; Rabinovici et al., 2008). Consistent with this and in a parallel theme in the literature, an atypical language variant of AD overlapping closely with the LPA syndrome has been described, largely based on retrospective correlation with post-mortem data (Alladi et al., 2007; Galton, Patterson, Xuereb, & Hodges, 2000). In the face of this strong association with AD, other studies have shown that patients with LPA may have TDP-43 pathology (Mesulam et al., 2008) suggesting that the clinico-pathological correlation of LPA with AD should not be considered universal. A key recent finding has been the discovery that mutations in the progranulin (*GRN*) gene can cause FTLD and in particular PPA (Baker et al., 2006; Cruts et al., 2006; Mesulam et al., 2007; Snowden et al., 2006, 2007b). Early descriptions suggested that these patients had a “non-fluent aphasia”: detailed case studies have described progressive anomia without motor speech impairment and subsequent development of repetition and reading deficits (Snowden & Neary, 2003; Snowden et al., 2007b). Based on the documented association of LPA with TDP-43 pathology (Mesulam et al., 2008) the phenotypic range of *GRN* mutations might also include LPA-like syndromes, however the true nosological place of LPA within the PPA spectrum and the core features of the LPA syndrome and the aphasic syndrome(s) that accompany *GRN* mutations have not been clarified.

Here we present a detailed clinical, neuropsychological and linguistic analysis of the language syndrome exhibited by a patient with a *GRN* mutation who presented with PPA. Our motivation for undertaking this study was twofold. Firstly, we wished to characterise the *GRN*-associated PPA syndrome in detail, and to assess the extent to which it is similar to or diverges from other PPA clinical syndromes: this speaks to the important nosological issue of commonality and diversity within the PPA spectrum. Secondly, we wished to put on record a new case with the neurolinguistic signature of a defined molecular lesion: this speaks to the broader issue of ‘molecular network-opathies’ in neurodegenerative disease (Rohrer et al., 2008b; Seeley, Crawford, Zhou, Miller, & Greicius, 2009).

## 2 Clinical details

A 62-year-old right-handed male retired shopkeeper, GAA, presented with a 3-year history of progressive word-finding difficulty. He would break off in mid-sentence, unable to find the words to finish, and would often say the opposite of what he meant (e.g., ‘yes’ for ‘no’, ‘left’ for ‘right’, ‘small’ for ‘big’). His speech became very sparse and he would overuse stereotyped phrases such as ‘at some stage’ and ‘it's aggravation’. He had difficulty repeating things told to him, understanding complex instructions and remembering messages. Early on in the illness he developed problems with arithmetic and subsequently also with reading, writing and spelling. He had no other cognitive symptoms. However, his family had noted he had become more socially withdrawn in recent years and less motivated. There was no family history of dementia in his parents (his mother died at the age of 80 of cancer and his father died at 70 of cardiac disease) however two of his mother's sisters developed dementia in their 80s and his mother's father had died after some time in a psychiatric hospital.

On examination he scored 19/30 on the MMSE (Folstein, Folstein, & McHugh, 1975) and 13/18 on the Frontal Assessment Battery (Dubois, Slachevsky, Litvan, & Pillon, 2000). There was mild bilateral ideomotor and ideational limb apraxia. The general neurological examination was unremarkable. He had a Clinical Dementia Rating (CDR)—total of 0.5 and CDR—sum of boxes of 4.0 (Morris, 1993). On a behavioural assessment, his total Neuropsychiatric Inventory score (Cummings et al., 1994) was 13, scoring 6 on depression/dysphoria, 2 on anxiety, 3 on apathy/indifference and 2 on irritability/lability subscales.

**Fig. 1:**
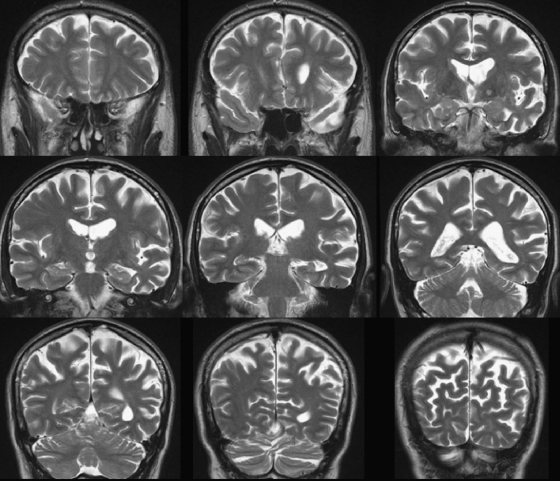
Coronal FLAIR magnetic resonance sections of the patient's brain (left hemisphere shown on the right) 3 years after symptom onset, showing predominant left fronto-temporo-parietal atrophy.

Brain MRI was performed 3 years after symptom onset (Fig. 1). This showed asymmetric atrophy predominantly involving the left cerebral hemisphere and accentuated in the temporal lobe (particularly the superior and lateral temporal cortex) and parietal lobe (supramarginal and angular gyri) with additional left prefrontal lobe atrophy. Changes of cerebrovascular disease were minimal. Following this study, he required a permanent pacemaker for cardiac conduction disease, precluding serial MR imaging.

A blood sample was obtained as part of a study into the genetics of young-onset dementia. All 13 exons of the *GRN* gene were sequenced in at least 1 direction. Analysis of electropherogram traces revealed the Arg493X mutation, the most common *GRN* mutation reported to date (Rademakers et al., 2007).

Neuropsychological and neurolinguistic functions were investigated in detail between 36 and 42 months following symptom onset.

## 3 General neuropsychology

There was a large discrepancy between GAA's very impaired verbal IQ score and average performance IQ score (on WAIS-III, Wechsler, 1981) (see Table 1). He was tested on four separate tests from The Camden Memory Tests battery (Warrington, 1996): his performance was below the 5th percentile on a test of verbal memory whereas visual memory was intact (10th to 25th percentile on a recognition memory test for faces, 95th percentile on a topographical recognition memory test and an errorless performance on a pictorial recognition memory test). Executive functions were relatively intact on two separate tests and performance was normal on tests of visuoperceptual and visuospatial skills (Warrington & James, 1991). However he was unable to score on the graded difficulty calculation test (Jackson & Warrington, 1986).

## 4 Speech assessment

### 4.1 Propositional speech

GAA's propositional speech was gravely impaired. He volunteered little spontaneous speech. At his first clinical assessment he was asked to describe his last holiday:

“I went to… the USA… for… *(long pause)* Boston… round there… we did round there… *(long pause)* we *you-sted* the… *(long pause)* all.” (48 seconds)

When asked to describe the Cookie Theft Scene from the Boston Diagnostic Aphasia Examination (Goodglass & Kaplan, 1983) he volunteered:

“This is falling out… they wanted that… they falling that… this was water… *(long pause)* that's about it I think… this was… this was along there… that's about it.” (30 seconds)

Analysis of these two short samples of spontaneous speech (total time 1.3 min) revealed a speech rate of 33 words/minute (in nine cognitively normal male controls with mean age 68, who spoke for an average of 2.6 min, the range was 102–148 words/minute). The mean log frequency of the words (based on the CELEX database, Baayen, Piepenbrock, & van Rijn, 1993) used was 3.41 (control range 2.24–2.73), mean log frequency of nouns (also based on CELEX database) used was 2.58 (control range 1.63–1.97) and noun imageability (based on the MRC database) was 596 (control range 509–574). There were no features of speech apraxia and the speech diadochokinetic rate was normal (Apraxia Battery for Adults-2 subtest 1: Dabul, 2000). There were relatively few speech production errors although there were rare phonemic and semantic errors. Although GAA's spontaneous speech was sparse and assessment for the presence of agrammatism was therefore difficult, there were nevertheless occasional clearly agrammatic errors, e.g., “we did round there” and “they falling that”. GAA was unable to perform sentence completion tasks of either high or low probability where he was given a sentence frame (e.g., he loosened the tie around his…) and asked to complete it with a single word (i.e., neck). On a second assessment 6 months after the initial assessment, GAA's spontaneous speech was even more severely impoverished—attempting to describe his last holiday he said:

“It's aggravation… *(long pause)* it's… can’t do the… *(long pause)* along there… can’t do… it's aggravation” (45 seconds)

Describing the Cookie Theft picture he said:

“That along there… along there, that's… that's…*(long pause)* see I don’t these… *(long pause)* I know what it is but I can’t do it, you know, it's aggravation” (35 seconds)

## 5 Detailed linguistic assessment

### 5.1 Naming

GAA was severely anomic scoring below the 1st percentile on the Graded Naming Test (McKenna & Warrington, 1980) (see Table 2). On a category naming test comprising high frequency nouns (Crutch, Randlesome, & Warrington, 2007) he had more difficulty with body parts than with animals, objects or colours. On a test comparing the naming of nouns (objects) and verbs (action pictures) matched for frequency using the CELEX database, performance was more impaired for verbs than nouns (*χ*^2^ = 4.33, *p* = 0.04). On analysis of errors made, he would commonly provide no answer, but when attempting an answer made mainly phonemic errors (e.g., ‘cheet’ for sheep; ‘flad’ for flag, ‘theeze’ for tweezers) and only occasional semantic (descriptive) errors (e.g., ‘red bits’ for bird (robin)).

### 5.2 Speech repetition

GAA's repetition of both single words and sentences was impaired (Table 2). He was able to repeat 78/120 words from a list comprising high and low frequency words and words of one, two or three syllables. Single word repetition showed a small but non-significant frequency effect (43/60 high frequency; 35/60 low frequency, *χ*^2^ = 2.34, *p* = 0.13) and a significant effect of syllable length (31/40 one-syllable words; 28/40 two-syllable words; 19/40 three-syllable words, *χ*^2^ = 8.57, *p* = 0.01) (see Table 2). Analysis of the 42 repetition errors revealed 11 items with no response (26%) and 31 phonological errors (11 substitutions (26%), 11 omissions (26%), 3 additions (7%), 1 transposition (2%) and 5 with multiple errors). GAA was able to repeat only 13/20 nonsense words. Sentence repetition was severely impaired: he was unable to repeat any of 10 short sentences or 10 clichés. In general he provided no response, however examples of errors made included:

**Table:** 

IT WAS TOO HOT	too hot
DEAF AS A POST	deaf as a front

### 5.3 Single word comprehension

GAA's performance was assessed on a series of single word comprehension tests, some of which involved direct matching between a word and target, and other tests which involved a degree of associative or inferential knowledge; performance was also compared on the visual versions of the associative tasks. GAA showed evidence of dissociated performance on these comprehension tasks (see Table 3). Thus his performance on the verbal (spoken and written input) version of the Pyramids and Palm Trees test (Howard & Patterson, 1992) was impaired, and furthermore significantly inferior to his performance on the visual version of the task which was within the normal range (sign test: *N* = 11, *x* = 2, *p* = 0.03). Similarly he had difficulty on the verbal version of the Camels and Cactus test (Bozeat, Lambon Ralph, Patterson, Garrard, & Hodges, 2000) compared to his normal score on the visual version (sign test: *N* = 17, *x* = 4, *p* = 0.03). GAA also attempted the short version of the British Picture Vocabulary Scale (Dunn, Dunn, Whetton, & Pintilie, 1982), and he scored below the 5th percentile with both written word and spoken word presentation. By contrast, on a test of semantic knowledge that probed attributes of size and weight in animals and objects respectively (Warrington & Crutch, 2007) he scored at a normal level on both the verbal and visual versions of the test. He was also assessed on the Category Specific Names Test assessing single word comprehension (McKenna, 1998): this test comprises arrays of five pictures selected from four categories, graded in difficulty so that the range of items encompasses very low frequency objects: on each section of this test (both spoken and written name to picture matching), he scored above the average level. He also attempted four graded two-choice (spoken and written) synonym comprehension tests, involving concrete and abstract nouns and verbs (Manning & Warrington, 1995; Warrington, McKenna, & Orpwood, 1998). He was clearly impaired on both the verb versions of the test (scores near chance) but within the normal range for both concrete and abstract nouns. He performed well on the Graded Naming Test presented as a forced three-choice recognition task in which he was presented simultaneously with a spoken and written definition for each item (e.g., “What is the large canvas covered frame upon which children can bounce and jump? – TARPAULIN, TAMBOURINE or TRAMPOLINE”).

### 5.4 Sentence comprehension and grammar

GAA's performance was below the 5th percentile on the Test of Reception of Grammar (TROG, Bishop, 1989). On a further set of 24 sentences taken from PALPA55 (Kay, Lesser, & Coltheart, 1992) his performance was significantly worse on reversible than nonreversible sentences and on passive than active sentences. Furthermore, performance did not benefit from a semantic variable (directionality). We explored GAA's comprehension of verb tense using an adapted version of the Lesser/Pizzamiglio and Parisi syntax test (Lesser, 1974; Parisi & Pizzamiglio, 1970) comprising 20 pairs of pictures which differ in whether the agent is doing something/has done something (present/past comparison, 10 items) or whether the agent is doing something/is about to do something (present/future comparison, 10 items). He scored 16/20 on this task scoring equally on the present/past and present/future items (healthy controls score at or near ceiling on this test). GAA was also tested on a grammaticality judgment test which was an adapted version of the test for syntactic abilities (Quigley, Steinkamp, Power, & Jones, 1978): this test entails a two-alternative forced choice on two sentences (presented simultaneously both visually and aurally), one of which is grammatical and the other agrammatical. The agrammatical sentences contained a variety of errors including incorrect verb tense, addition/substitution/deletion of function words and incorrect word order. GAA scored 79% on this test making 23 errors of which 15 were errors made on incorrect verb tense (see Table 3).

### 5.5 Reading

GAA was able to read single letters fairly competently with only 1 error from 25 letters (see Table 4). However he had great difficulty reading both real words and nonwords (Snowling, Stothard, & McLean, 1996). Investigating his real word reading further, he had similar difficulty in reading regular and irregular words. He had greater difficulty with abstract words than concrete words and with increasing word length. A battery of 275 three-letter words was also administered to examine reading errors: he read 77% of the words correctly, with 62 errors in total. Included in this test were 55 three-letter function words: there were errors on 29% of these words (compared to 21% errors on the other 220 content words). There was a mixture of error types across the reading subtests, comprising mainly phonological (e.g., ‘opperosite’ for opposite) and visual (e.g., ‘December’ for decent) errors but also occasional regularisation (e.g., ‘gem’ with hard ‘g’ for gem), and semantic errors (e.g., ‘salt’ for sour).

### 5.6 Writing and spelling

GAA's spelling was severely impaired. He was unable to score on the written graded difficulty spelling test (Baxter & Warrington, 1994) (see Table 4). His attempts for the first four items were ‘ONE’ for TWO, ‘BULL’ for WORLD, ‘SEA’ for SAID and ‘NICE’ for SHOE. On a further set of three-letter words he scored equally poorly on both regular and irregular words and oral and written spelling were comparably affected. He made seven errors on oral spelling, comprising five no responses and the errors ‘SIK’ for SEA and ‘SAT’ for CAP; six errors on written spelling, comprising single letters: ‘S’ for SON and SAW, ‘M’ for CUP, ‘W’ for LOG and ‘M’ for BAR. On attempting to write single letters to dictation he was able to produce only 5 of 25 letters.

GAA was asked to construct grammatical sentences containing each of 10 written target words. He made no attempt for three words (‘new’, ‘radio’, ‘tree’), and for the remaining seven words produced the following:

**Table:** 

EARLY	Early a clock eight
CAUGHT	Caught a sam
PUSHED	Pushed on door
SMALL	Small emp
WALKED	Walked a patio
THROW	Throw on door
BLUE	Blue door

#### 5.6.1 Short-term memory

GAA's digit span, assessed as part of the WAIS-III, was severely impaired (see Table 5). We subsequently compared his auditory–verbal digit span, auditory–verbal letter span, auditory–verbal word (three-letter, one-syllable) span, visual–verbal digit span and spatial span. In each condition, eight trials were presented with one, two or three items. GAA was unable consistently to repeat more than one item for spoken digits, letters or words. Performance was better for visually presented digits, for which he was occasionally able to repeat three items. Furthermore, in stark contrast to his performance on the auditory tasks, his spatial span (assessed with the Corsi block-tapping test, Corsi, 1972; Kessels, van Zandvoort, Postma, Kappelle, & de Haan, 2000) was within the normal range—he was able to point without error to three blocks, scored 4/8 completely correct trials (24/32 positions) with four blocks and 1/8 completely correct trial (22/40 positions) with five blocks.

## 6 Discussion

Here we have described in detail the pattern of neuropsychological and linguistic deficits in a patient with *GRN*-associated PPA. The salient clinical features were sparse, slow and impoverished spontaneous speech with word-finding pauses. The profile of neuropsychological deficits comprised severe anomia, poor verbal short-term memory and impaired sentence comprehension, associated with dyslexia, dysgraphia and dyscalculia. By contrast certain (non-associative) aspects of single word comprehension, non-verbal memory and visual perceptual skills were well preserved. The constellation of neuropsychological findings in GAA constitutes a distinctive pattern of cognitive impairment and preservation. The clear verbal modality specificity of GAA's language deficits indicates preferential involvement of the dominant hemisphere, while the association of dyslexia, dysgraphia and dyscalculia constitutes a classical left parietal syndrome; the lobar localisation for other features, such as anomia and impaired phonological memory, is less clear. This neuropsychological syndrome overlaps in a number of respects with previous descriptions of the LPA syndrome (Gorno-Tempini et al., 2008) while the presence of grammatical errors in spontaneous speech and markedly impaired speech repetition suggests an additional overlap with the PNFA syndrome. However, the cognitive profile exhibited by GAA should not be regarded simply as a variant or a composite of other PPA syndromes: key features of this profile in relation to LPA and PNFA are summarised in Table 6. Anatomically, although detailed correlation was not possible, cerebral atrophy in this case involved the left posterior temporal/anterior parietal region and also left inferior frontal areas (Fig. 1). According to the current dual stream model of cortical language processing, a ventral pathway involved in processing word meaning links the superior temporal gyrus to middle and inferior temporal gyri, temporal pole and inferior frontal cortex; while a dorsal pathway involved in articulation-to-sound mapping links the superior temporal gyrus with inferior parietal and inferior frontal cortices (Hickok & Poeppel, 2004; Saur et al., 2008; Warren, Wise, & Warren, 2005). Following this formulation, and taking the neuropsychological and neuroimaging evidence into account, we propose that *GRN*-associated PPA in this case is likely to reflect involvement of both the dorsal and ventral language pathways, with a key site of overlap in the region of the temporo-parietal junction. We now consider the evidence for this claim in more detail.

GAA had progressive anomia. While this is likely to be attributable at least in part to impaired word retrieval, a verbal semantic deficit may also have contributed. The pattern of GAA's performance on single word comprehension tests is relevant both to neuropsychological theories of semantic knowledge as well as how such a syndrome would fit into current PPA classifications. He had no difficulty with the Size/Weight Attribute Test of conceptual knowledge and more impressively he scored at a high level on both the spoken and written word versions of the Category Specific Names Test probing knowledge of low frequency items. Furthermore, on a synonyms test of concrete noun comprehension his performance was at an average level. By contrast, on word–picture matching tests such as the British Picture Vocabulary Scale where the mapping between word and target picture is less direct, his performance was impaired. He was also impaired on verbal (spoken and written word to word) matching tasks such as the verbal versions of the Pyramids and Palm Trees and Camels and Cactus tests whilst exhibiting normal performance on the visual versions. How can we explain the profile of dissociated verbal semantic impairments observed in GAA?

Considering the noun comprehension tests, we suggest that GAA's weaker performance is observed on those tasks involving some degree of associative (or inferential) rather than direct semantic processing. Associative tasks are likely to involve executive control processes, as suggested by Jefferies and Lambon Ralph (2006). However, a primary deficit in executive control would not easily explain the difference between GAA's performance on verbally and visually mediated versions of these associative tasks. This visual advantage is in contrast to the pattern of performance described in stroke patients (Corbett, Jefferies, Ehsan, & Lambon Ralph, 2009; Jefferies & Lambon Ralph, 2006), and belies the equal semantic control demands of the visual and verbal versions of this task. Another possibility is that GAA has mildly impaired lexical semantics, such that response selection among closely related alternatives is required to expose degraded semantic representations; or alternatively, an intact semantic store but a deficit in linking phonological representations of words with their meanings, which is exposed when the semantic targets are closely related. Picture–picture matching might provide additional information or cues unavailable from the written or spoken word, with correspondingly better performance on visual than verbal matching tasks. An explanation of this kind would be in line with evidence from studies of focal lesions such as stroke affecting associative cortical areas in the region of the temporo-parietal junction (Hillis, 2007). Moreover, degraded access to verbal semantic stores resulting from posterior temporal-inferior parietal lobe atrophy would be consistent with functional imaging evidence in healthy subjects suggesting that the extraction of meaning from both spoken and written language may require connectivity between posterior and anterior temporal lobe areas in the ventral language stream (Spitsyna, Warren, Scott, Turkheimer, & Wise, 2006).

A test such as Pyramids and Palm Trees seems to call for manipulation of concepts and contexts (e.g., in order to decide whether “cat” or “dog” is the correct answer when presented with “mouse”, one must not only comprehend individual concepts but also activate the salient relationships between target and response, i.e., “hunter/hunted” rather than “both animals” or “do not bark”, etc.). We therefore raise the further possibility that the dissociation between verbal and non-verbal comprehension performance observed in GAA may arise from a selective deficit of verbal reasoning. ‘Verbal reasoning’ is itself an under-specified term: we use it here to embrace several potentially relevant processes, in particular inference or abstraction of a semantic relationship that is not directly implied by the stimuli. That such processes can be specific to the verbal modality is supported by the existence of a selective deficit of verbal message formulation in patients with so-called “dynamic aphasia” (Costello & Warrington, 1989; Warren, Warren, Fox, & Warrington, 2003). The present study does not disambiguate any deficit in verbal reasoning from a mild deficit of lexical semantics (indeed, that distinction is difficult even in principle). However, processes such as verbal inference are likely to involve fronto-parietal circuitry (Reverberi et al., 2007), raising the possibility that the associative verbal semantic deficit we have identified in GAA might implicate either the dorsal or the ventral language pathway (or indeed, a conjoint deficit attributable to temporo-parietal junction damage).

In detailed descriptions of LPA, patients have performed well on the visual version of the Pyramids and Palm Trees test, leading to the suggestion that semantic memory is intact in patients with LPA (Gorno-Tempini et al., 2004, 2008). The present evidence suggests a qualification of this position, in that at least some patients may have intact performance on this visual test, yet still perform poorly on certain other tests of single word comprehension, in particular those requiring associative or inferential verbal semantic processing. It is unclear whether this is a distinct feature of a *GRN*-associated PPA syndrome or an effect of disease progression or worsening severity in the LPA syndrome where progressive left hemispheric atrophy encroaches on posterior semantic areas. There is some evidence that patients with LPA show impaired single word comprehension with disease progression and the overlapping pattern of anatomical involvement of the temporo-parietal junction in both LPA associated with AD and *GRN* mutations suggests that this may be a feature of the neuroanatomy rather than the underlying molecular substrate (Beck et al., 2008; Gorno-Tempini et al., 2008; Rabinovici et al., 2008). More detailed longitudinal studies of LPA and *GRN* disease progression will be necessary to investigate this further.

GAA showed evidence of an impaired phonological store (poor verbal short-term memory). His auditory–verbal span was not entirely intact even for single items (digits, letters or words), while visual–verbal span was only marginally better. This contrasted with his normal visuospatial span. In addition, GAA's performance was impaired on tests not only of receptive grammar (e.g., TROG, PALPA55) but also grammaticality judgement tests (e.g., test of syntactic abilities). Previous evidence suggests that although they may cause deficits in sentence comprehension tasks, auditory–verbal span deficits are neither necessary nor sufficient to produce such deficits in receptive grammar and grammaticality judgements (e.g., Caplan & Waters, 1999; Shallice & Butterworth, 1977). We propose that GAA has a double deficit affecting both his auditory–verbal short-term memory and the systems mediating receptive grammar. This would also be consistent with the distributed pattern of left cerebral atrophy with left temporo-parietal emphasis in this case: the phonological store is likely to be mediated by anterior inferior parietal and posterior superior temporal areas whilst sentence and grammatical processing are associated with inferior frontal and posterior superior temporal areas (Buchsbaum & D’Esposito, 2008; Vigneau et al., 2006). Sentence comprehension has been studied in LPA with suggestions that deficits are secondary purely to phonological store deficits (Gorno-Tempini et al., 2008). However, there have been no previous studies attempting to dissociate a true receptive grammatical deficit from a phonological store deficit in LPA (e.g., on a grammaticality judgment test). Similarly, it has been difficult to characterise any expressive agrammatism in LPA, as speech tends to be sparse with prolonged pauses. In this study there was some evidence for agrammatism in GAA's spontaneous speech and further evidence in his production of very simple or agrammatic sentences in writing. This may represent a further distinction from the LPA syndrome (suggesting an overlap with the classical PNFA syndrome), but again, will require further study, particularly with detailed quantitative analysis of spontaneous speech and writing in this group.

With further regard to his deficit of receptive grammar processing, GAA had particular difficulty with comprehension of verb tense which, in conjunction with poor performance on verb naming and verb comprehension tasks, suggests a relatively selective deficit of verb (versus noun) processing. Anatomically, verb processing is thought to rely on left dorsal language pathway areas including left prefrontal cortex (Damasio & Tranel, 1993) and posterolateral temporal cortex (Grossman et al., 2002), consistent with the pattern of atrophy seen here. Of note, a selective deficit in verb processing has been previously described in a familial ubiquitin-positive inclusion dementia (Bak et al., 2006): although the genetic diagnosis in this previous case was not defined, considered together these observations raise the possibility that defective verb processing may be a signature of *GRN* mutations in PPA.

GAA exhibited additional deficits of literacy skills that provide further evidence of deficient phonological processing. His reading deficit shows the typical pattern of deep/phonological dyslexia affecting regular and irregular real words as well as nonwords, the errors produced being a mixture of phonological, visual and more rarely regularisation and semantic errors, with better performance reading concrete compared to abstract words (Coltheart, 1980; Crisp & Lambon Ralph, 2006). Similarly his pattern of spelling deficits indicates phonological dysgraphia in both oral and written modes. The presence of phonemic errors would be consistent with a deficit of phonological transcoding, which may result from damage to the left temporo-parietal junction. Patients with LPA have previously been described as having phonological dyslexia (Brambati, Ogar, Neuhaus, Miller, & Gorno-Tempini, 2009) and a more general deficit of phonological processing (Gorno-Tempini et al., 2008).

It is worth considering how this neurolinguistic and anatomical formulation may relate to other clinical features in this case and in previous descriptions of *GRN*-associated disease. GAA did not exhibit neurological signs of parkinsonism (described in around a third of *GRN* mutation cases) or motor neurone disease (a rare feature) (Baker et al., 2006; Beck et al., 2008; Cruts et al., 2006). However, GAA did display evidence of apathy and depression as well as increased anxiety and irritability: such behavioural changes have been previously reported with *GRN* mutations (Beck et al., 2008; Snowden et al., 2006) and indeed, the most common clinical phenotype of *GRN* mutations is progressive personality change (behavioural variant frontotemporal dementia). Similar behavioural symptoms have been described in association with both PNFA and LPA (Rosen et al., 2006). In anatomical terms, such complex behaviours are likely to depend on distributed circuitry and might therefore be vulnerable to disease processes that strike long intra-hemispheric pathways linking frontal and anterior temporal cortices with more posterior areas, as we propose may underpin the *GRN*-associated aphasic syndrome here.

Beyond demonstrating a molecular and anatomical association, aphasia associated with *GRN* mutations suggests a pathophysiological mechanism that may underpin certain key features of the LPA syndrome. Broadly, a number of features can be understood as the consequence of breakdown of phonological processing due to dysfunction of the left temporo-parietal junction and its connections. However, this case has highlighted certain neuropsychological differences with respect to previous descriptions of the LPA syndrome (Brambati et al., 2009; Gorno-Tempini et al., 2004, 2008, see Table 6), in particular, the early occurrence of single word comprehension deficits (also a feature in our previously described case of *GRN*-associated PPA, Rohrer et al., 2008b) and receptive (and also mild expressive) agrammatism. Detailed longitudinal single case analysis of *GRN*-associated FTLD has shown a strikingly asymmetric pattern of involvement of functionally connected but distributed cortical areas within a cerebral hemisphere (Rohrer et al., 2008b), and the finding of markedly asymmetric left temporal and parietal atrophy in the present case would be consistent with this. Involvement of the key left temporo-parietal junction zone is predicted to correlate with involvement of functionally connected regions in the left inferior frontal and anterior temporal lobes via the dorsal and ventral speech processing pathways demonstrated in functional imaging studies in healthy subjects (Scott & Johnsrude, 2003) and implicated in clinical aphasia syndromes of vascular disease (Hillis, 2007; Jefferies & Lambon Ralph, 2006; Rohrer et al., 2008a). Any conclusions based on the detailed analysis of a single case must be extrapolated with care, in order to assess their relevance to the wider population of patients with the syndrome. The key unresolved issue raised by this case is whether the features here typify a discrete syndrome of *GRN*-associated aphasia, or rather, one instance of a broader continuum of non-fluent aphasia cases with different molecular substrates. It will be important to conduct further group and detailed single case studies in patients with *GRN*-associated PPA to define the full clinico-pathological and clinico-genetic spectrum of the disorder, to establish the extent to which *GRN*-associated PPA, AD-LPA and other non-fluent cases can be distinguished on neuropsychological grounds, and to address in detail the anatomical and pathophysiological basis of the *GRN*-associated language ‘network-opathy’.

## Figures and Tables

**Table 1:** General neuropsychological assessment.

Test	Score	Percentile score
General intelligence		
WAIS-III verbal IQ	53	
WAIS-III performance IQ	102	

Episodic memory		
Short Recognition Memory Test for words	18/25	<5th
Short Recognition Memory Test for faces	20/25	10–25th
Topographical Recognition Memory Test	28/30	95th
Pictorial Recognition Memory Test	30/30	>10th%

Executive function		
Trail making test A scaled score	7	10–25th
Trail making test B scaled score	10	50th
D-KEFS design fluency composite scaled score	8	10–25th

Visuoperceptual/visuospatial skills		
Visual object and space perception battery (VOSP) test 3—object decision	19/20	>75%
VOSP test 5—dot counting	10/10	>5%

Arithmetic		
Graded difficulty calculation test	0/24	<5th

**Table 2:** Detailed linguistic assessment: naming and speech repetition.

Test	Score	Percentile score/normal range (NR)
Naming		
Graded Naming Test	4/30	<5th
Category naming test	23/40	
Animals	7/10	NR 8–10
Objects	6/10	NR 10
Colours	7/10	NR 9–10
Body parts	3/10	NR 10

Matched noun and verb naming test		
Nouns	6/20	NR 18–20^a^
Verbs	1/20	NR 18–20^a^

Speech repetition		
Single words^b^	78/120	c
Nonwords	13/20	c
Short sentences	0/10	c
Cliches	0/10	c

a Normal range based on a cognitively normal control sample of 18 patients (9 male, 9 female) with an average age of 67.9.

b See text for detailed analysis.

c Normal controls are assumed to be at ceiling on these tasks.

**Table 3:** Detailed linguistic assessment: comprehension of single words, sentences and grammar.

Test	Score	Percentile score/normal range (NR)
Single word comprehension		
Pyramids and Palm Trees test		
Verbal^c^ (three words)	43/52	NR 49–52
Visual (three pictures)	50/52	NR 49–52

Camels and Cactus test		
Verbal^c^ (five words)	46/64	NR 56–63
Visual (five pictures)	55/64	NR 51–62

British Picture Vocabulary Scale (short)		
Written word to picture matching	21/32	<5th
Spoken word to picture matching	24/32	<5th

Size/Weight Attribute Test		
Verbal animals	30/30	NR 26–30
Visual animals	29/30	NR 27–30
Verbal objects	27/30	NR 26–30
Visual objects	29/30	NR 26–30

Category Specific Names Test		
Written presentation		
Fruit	30/30	Control mean 25.0^b^
Animals	30/30	Control mean 28.3^b^
Praxic objects	26/30	Control mean 29.2^b^
Non-praxic objects	30/30	Control mean 26.8^b^

Spoken presentation		
Fruit	25/30	Control mean 24.8^b^
Animals	30/30	Control mean 28.2^b^
Praxic objects	30/30	Control mean 29.2^b^
Non-praxic objects	29/30	Control mean 26.7^b^

Warrington synonyms test^c^		
Concrete nouns	21/25	50–75th
Abstract nouns	18/25	10–25th
Concrete verbs	15/25	Control mean 22^d^
Abstract verbs	15/25	Control mean 20^d^

Graded Naming Test from description (forced choice of three words)^c^	23/30	

Sentence comprehension and grammar		
Test for the Reception of Grammar (TROG)	45/80	<5th
PALPA 55 (modified version)	17/24	NR 22–24^a^
Reversible	63%	
Non-reversible	88%	
Passive	58%	
Active	83%	
Directional	50%	
Non-directional	75%	
Verb tense comprehension test	16/20	NR 19–20^a^
Test of syntactic abilities (modified)	85/108	

a Normal range based on a cognitively normal control sample of 18 patients (9 male, 9 female) with an average age of 67.9.

b Based on control sample of 10 subjects from McKenna and Parry (1994).

c Presented simultaneously in both spoken and written form.

d Based on control sample of three subjects from Manning and Warrington (1995).

**Table 4:** Detailed linguistic assessment: literacy skills.

Test	Score^a^
Reading	
Single letter reading	24/25
National Adult Reading Test	0/50 (<1st%)
Graded difficulty nonword reading test	2/20 (<10th%)
Coltheart irregular vs regular word reading test	31/78
Irregular words	15/39
Regular words	16/39
Concrete/abstract reading test	47/72
Abstract words	18/36
Concrete words	29/36
High frequency words	23/36
Low frequency words	24/36
1 syllable length	21/24
2 syllable length	17/24
3 syllable length	9/24

Writing/spelling	
Sentence construction	0/10
Graded difficulty spelling test	0/30 (<1st%)
Three-letter word spelling test	7/20
Regular words	5/10
Irregular words	2/10
Oral spelling	3/10
Written spelling	4/10
Single letter writing	5/25

a All cognitively normal adults score at a ceiling level on tests apart from the NART, graded difficulty nonword reading test and graded difficulty spelling test.

**Table 5:** Short-term memory assessment.

Task	One item	Two items	Three items
Auditory–verbal digit span	6/8	1/8 (5/16)	Unable

Auditory–verbal letter span	7/8	Phonologically similar 1/8 (6/16)	Unable
		Phonologically dissimilar 0/8 (3/16)	

Auditory–verbal word span	5/8	0/8 (3/16)	Unable
Visual–verbal digit span	7/8	6/8 (14/16)	2/8 (15/24)
Spatial span	8/8	8/8 (16/16)	8/8 (24/24)

Eight stimuli for each task at each level—scores are shown as total completely correct/8 and in parentheses the total number of items in the correct position (16 for 2 items and 24 for 3 items).

**Table 6:** Comparison of neuropsychological features in GAA compared to previous LPA and PNFA cases.

Neuropsychological feature	GAA	LPA	PNFA
Spontaneous speech	Slow, sparse spontaneous speech with word-finding pauses	Slow spontaneous speech with word-finding pauses	Speech characterized by hesitancy and effortfulness due to apraxia of speech and/or agrammatism
Naming	Severely anomic	Anomic	Mildly anomic
Single word repetition	Moderately impaired	Relatively intact (compared to sentence repetition)	Mild to moderately impaired
Sentence repetition	Severely impaired	Impaired	Impaired
Single word comprehension	Impaired for associative/inferential verbal semantic tasks, verb comprehension; intact for direct semantic processing of nouns	Relatively intact	Intact early in the course
Sentence comprehension and grammar	Severely impaired, possible true grammatical deficit (expressive and receptive)	Impaired	Impaired
Reading	Deep/phonological dyslexia	Phonological dyslexia	Little studied but phonological dyslexia described
Verbal short-term memory	Severely impaired	Severely impaired	Usually intact early
Episodic memory	Impaired verbal, intact non-verbal	Few studies but evidence of mild verbal impairment	Intact
